# The Influence of the Thickness of the Materials for the Provisionalization in Minimally Invasive Restorations

**DOI:** 10.3390/ma15207238

**Published:** 2022-10-17

**Authors:** Ildefonso Serrano-Belmonte, Nerea Roca-Cánovas, Eva K. Linares-Tovar, Ascensión Martínez-Cánovas, Virginia Pérez-Fernández

**Affiliations:** 1 Faculty of Medicine, School of Dentistry, Clínica Odontológica Universitaria, Hospital Morales Meseguer, Universidad de Murcia, Avda. Marqués de los Vélez s/n, 30008 Murcia, Spain; 2 Departamento de Ciencias Sociosanitarias, Institute of Biomedical Research (IMIB-Arrixaca), Universidad de Murcia, 30120 Murcia, Spain

**Keywords:** BOPT technique, temporary materials, flexural strength, modulus of elasticity

## Abstract

This in vitro study aimed to determine the flexural strength and modulus of elasticity of two bisacrylic resins for temporary crowns at different thicknesses, i.e., Structur 3 and Structur Premium, and to compare them with each other. Sixty samples were prepared, thirty of each material, which were made at different thicknesses (1 mm, 1.5 mm, and 2 mm). The dimensions followed the UNE-EN ISO 178:2019 standard, with a length of 24 mm, a width of 10 mm, and the thicknesses described. Materials were subjected to a three-point bending test. For the modulus of elasticity, statistically significant differences were observed between the 1.5 mm and 2 mm thicknesses of Structur 3 material. For Structur Premium, statistically significant differences were observed between the thicknesses 1 mm and 1.5 mm as well as between 1 mm and 2 mm thickness. With respect to flexural strength, no statistically significant differences were observed for either material at the different thicknesses. Significant differences were observed between the materials for both flexural strength and modulus of elasticity, being higher for Structur Premium: Structur Premium has a higher flexural strength and modulus of elasticity than Structur 3. There are significant differences for the modulus of elasticity but not for the flexural strength between thicknesses.

## 1. Introduction

Minimally invasive dentistry (MIS) is a paradigm of dentistry today. Throughout history, a concern has arisen on the part of both patients and clinicians: the elimination of healthy dental tissue. Therefore, the aim is to maintain as much healthy dental tissue as possible, for which research continues today on new techniques and materials designed to meet this objective. For example, Loi and Di Felice described a new technique for tooth preparation and management of the provisional restoration by creating a new crown with a new emergence profile that simulates the shape of the natural tooth: the BOPT protocol. Healing over this restoration is a critical step to guide the gingival tissues to a new profile [1,2,3].

Likewise, new prosthetic restorative materials tend to be increasingly thinner, managing to maintain esthetics while improving their strength and ensuring good long-term performance, such as translucent monolithic zirconia ceramics, feldspathic porcelains, lithium disilicate glass-ceramics, or lithium silicate glass-ceramics with zirconia [4,5,6]. Therefore, in the face of increasingly smaller preparations, temporary materials are needed that meet these requirements of resistance at minimum thickness and that have ideal mechanical and physical properties, including resistance to bending, wear, and fracture [7,8]. It should also be considered that, in techniques such as BOPT, they should maintain their function for long periods of time and not only during the manufacturing process of the definitive prosthesis since they are essential for the formation of a new gingival profile from the first moment.

The materials used for these provisional prostheses can be classified into: powder-liquid systems (polymethacrylate/methylmethacrylate (PMMA/MMA) or higher molecular-weight methacrylates such as polyethylmethacrylate (PEMA) and paste-paste systems (bis-acrylic resins). Currently, bis-acrylic resins are the most frequently used. These bisacrylic composites stand out from PMMA materials in terms of their mechanical properties [9].

In the specific case of this study, based on determining the flexural strength of two temporary bis-acrylic materials, Structur 3 and Structur Premium (VOCO GmbH, Cuxhaven, Germany) were used. These can be used both for making provisional fixed restorations directly in the clinic (direct method) and for relining laboratory-made temporary crowns (indirect method).

To know the masticatory forces that a specific material is capable of resisting, it is necessary, among other things, to determine the flexural strength and the modulus of elasticity of the material, both of which are directly proportional.

The flexural strength test can be carried out by static or dynamic methods. Among the static methods, the test developed in this work includes the three-point bending test described in ISO 178:2019. 

Since the thicknesses of crowns are becoming smaller, at present, it is very important to analyze the influence of the thickness on the mechanical properties of the materials. New restorative materials in fixed prostheses as well as new techniques such as BOPT make it essential to study the properties of interim materials, which must respond to less thickness and longer periods of use in the tooth.

Therefore, the objective of this study is determining the flexural strength and modulus of elasticity of two bisacrylic resins for temporary crowns at different thicknesses, namely Structur 3 and Structur Premium, and to compare them with each other. The results could influence the selection of an appropriate product for patient treatment.

## 2. Materials and Methods

### 2.1. Specimen Preparation

For this in vitro study, based on a 3-point flexural strength test, 30 samples of Structur 3 Voco and another 30 samples of Structur Premium Voco (Cuxhaven, Germany) were prepared with different thicknesses: 1 mm, 1.5 mm, and 2 mm. The specimens made with both materials had dimensions of 24 mm long × 10 mm wide, following the UNE-En ISO 178:2019 standard: “Plastics. Determination of flexural properties”. This standard specifies the method to be carried out in order to determine the flexural properties of these materials used in the manufacture of temporary crowns. It defines a standard type of specimen but also includes certain parameters to be followed for the fabrication and use of alternative specimens. According to the standard, at least 5 specimens should be prepared, but in this case, more were prepared to obtain a greater accuracy in the study. 

Thus, 20 samples of 1 mm thickness were obtained (10 of Structur 3 and 10 of Structur Premium) as well as another 20 samples of 1.5 mm thickness (10 of Structur 3 and 10 of Structur Premium) and another 20 samples of 2 mm thickness (10 of Structur 3 and 10 of Structur Premium). 

**Figure 1:**
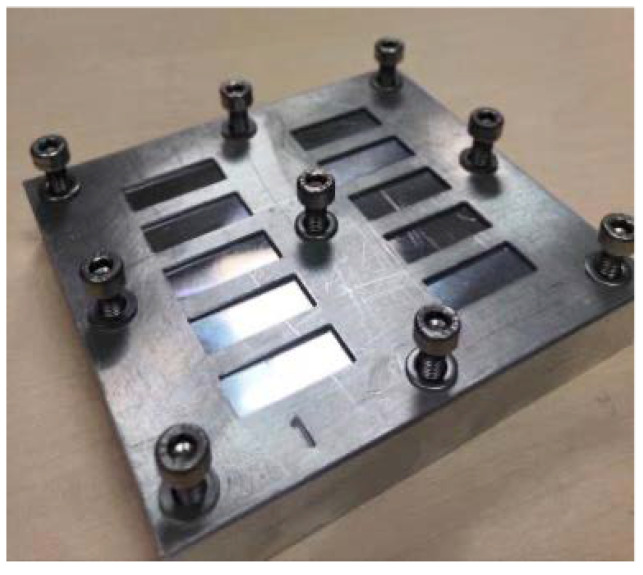
Metal well (1 mm thickness).

The specimens were made from metal wells made at the CAID (Research and Development Support Center) of the University of Murcia (Figure 1), thus being able to obtain the specific dimensions necessary to carry out the test. These dimensions were: a first group of wells of 24 × 10 × 1 mm, a second group of 24 × 10 × 1.5 mm, and a last group of 24 × 10 × 2 mm.

The preparation of the specimens took place in the Prosthesis Laboratory of the Clínica Odontológica Universitaria at the Morales Meseguer Hospital. Once the cartridge of material had been placed in the auto-mixing gun, the metal wells were carefully filled. Once they were filled, they were quickly flushed to ensure that they had the correct thickness. 

For this purpose, two metal plates made and designed at CAID helped to carry out this task quickly, comfortably, and accurately. These plates were placed on top of the wells filled with material, and pressure was exerted on them to remove all excess material. The procedure was repeated until all 60 samples were available (30 of Structur 3 and 30 of Structur Premium).

After all the specimens had been prepared, their thickness was checked using a thickness gauge.

**Figure 2:**
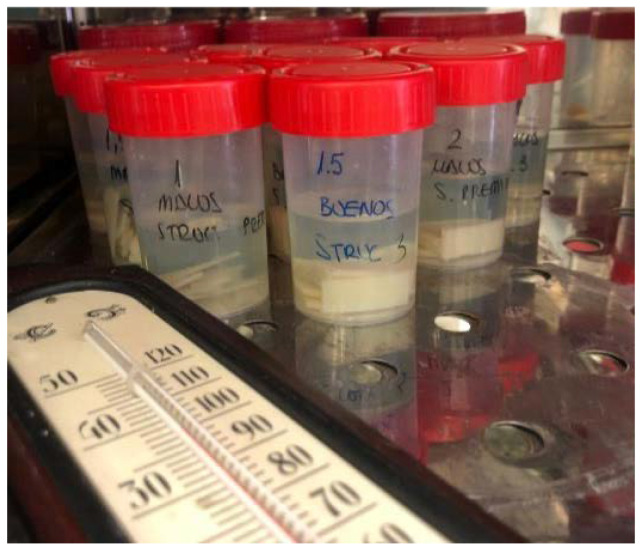
Immersion in artificial saliva.

The specimens were then stored for 28 days in transparent plastic jars containing artificial saliva. The solution selected to maintain the specimens was Xerostom mouthwash (Biocosmetics Laboratories, Madrid, Spain). They were divided into jars according to their different thicknesses. Once immersed in the jars with artificial saliva, they were placed in the culture oven at 38 °C until the test was performed (Figure 2).

### 2.2. Three-Point Bending Resistance Test

A Shimadzu machine, model AG-X PLUS (Kyoto, Japan), located at the LAIB (Biosanitary Research Laboratory) of the Hospital Virgen de la Arrixaca (Murcia, Spain), was used to carry out the procedure.

The test was carried out to determine the flexural strength of the materials used. Flexural strength can be defined as the measure of the resistance of an element or material to bending forces. It is the maximum bending stress that is withstood by the specimens. It is expressed in N/mm2 or megapascals (MPa) and is calculated from the following formula:

σfM = 3 Fd/2 wh2

F is the maximum applied force, in N;

d is the distance between supports, in mm;

w is the width of the specimen, in mm;

h is the thickness of the specimen, in mm.

**Figure 3:**
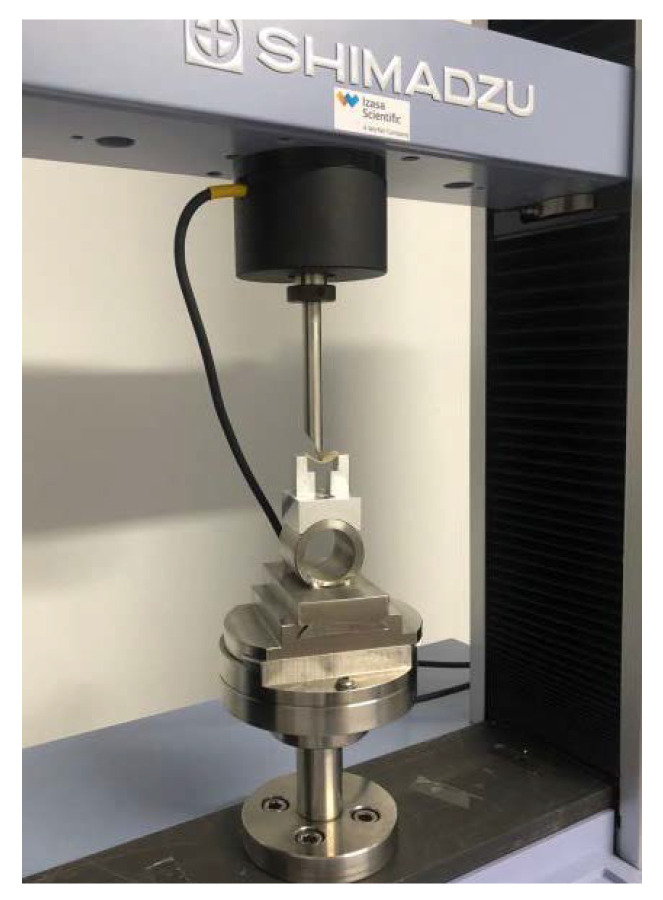
Bending of specimen.

For the test, the rectangular section specimens stood on two supports and were bent by a third element that acted on the midpoint of the specimen between the other two supports (Figure 3). 

The specimen was subjected to bending at a constant speed of 1 mm/min according to the standard ISO 178:2019, which indicates the appropriate speeds for the test to be performed. Specifically, it is the speed established by the standard for specimens of thickness between 1–3.5 mm.

The flexural strength test can be carried out by static or dynamic methods. Among the static ones was the test that was developed in this work: the 3-point bending test described in ISO 178:2019, as this is commonly used to simultaneously evaluate the flexural strength and the modulus of elasticity.

### 2.3. Data Collection

Once the samples were tested, the program provided a report with the results obtained, which was saved as a new document. From it, tables were prepared to be statistically analyzed. In addition, the data of the force exerted until breaking the specimen (maximum force) as a function of time and displacement were also collected and stored in a spreadsheet. Both documents, once collected and sorted, were statistically analyzed.

### 2.4. Statistical Analysis

#### 2.4.1. Descriptive Statistics

The following variables were calculated: mean and standard deviation, and the Kolmogorov–Smirnov test was performed to evaluate the normality of the variables.

#### 2.4.2. Inferential Statistics

Two variables were analyzed: bending and elasticity of the two materials, using repeated measures analysis for two factors (thickness and type of material). The statistical analysis was carried out with the non-parametric Mann–Whitney U test to compare elasticity and flexural strength of materials against each other and with the test of Kruskal–Wallis to compare elasticity and flexural strength between the different thicknesses for each compound. The significance level was established as *p* < 0.05.

The software Stata version 15.0 was used to carry out the statistical analysis.

## 3. Results

**Figure 4:**
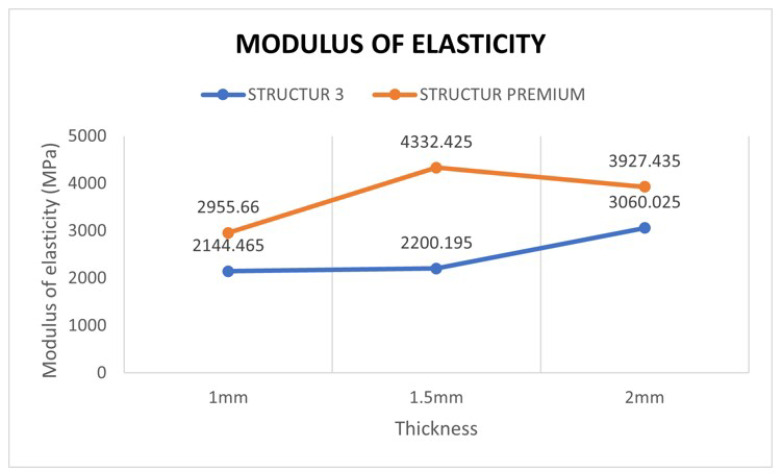
Modulus of elasticity obtained in both materials and comparison between different thickness (median).

When analyzing the data resulting from the comparison between the two materials with respect to the modulus of elasticity, it can be concluded that there were statistically significant differences (*p* < 0.001) (Figure 4). 

**Figure 5:**
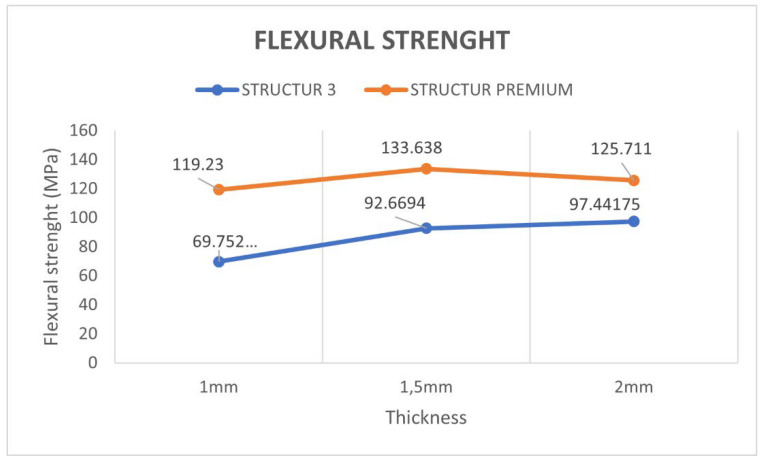
Flexural strength obtained in both materials and comparison between different thickness (median).

With respect to the data resulting from the comparison between the two materials with respect to flexural strength, results showed that there were statistically significant differences (*p* < 0.001) (Figure 5).

When comparing the modulus of elasticity of the Structur 3 material at three different thicknesses, no significant differences were found between thicknesses (*p* = 0.0718) (Table 1).

Regarding the modulus of elasticity of the Structur Premium material at three different thicknesses, it was observed that there were statistically significant differences between thicknesses (*p* = 0.0044) (Table 2).

When comparing the flexural strength of the Structur 3 material at three different thicknesses, no significant differences were found between thicknesses (*p* = 0.0568) (Table 3). 

Regarding the flexural strength of the Structur Premium material at three different thicknesses, no significant differences were found between thicknesses (*p* = 0.2537) (Table 4).

## 4. Discussion

To carry out the flexural strength test, the instructions of the UNE-EN ISO 178: 2019 procedure were followed. This standard has also been used by other works or studies, such as those of Zafra [8], Naresh [10], or Canals [11]. Other studies based the preparation of their samples with measurements of 25 × 2 × 2 mm, according American Dental Association specification No. 27 [12,13,14]. Other measurements used that deviate from UNE-EN ISO 178:2019 are 3 × 5 × 90 mm [15] and 65 × 10 × 3 mm [16]. For this study, 10 Structur 3 specimens of 1 mm thickness, 10 of 1.5 mm, and 10 of 2 mm and 10 Structur Premium specimens of 1 mm thickness, 10 of 1.5 mm, and 10 of 2 mm, all measuring 24 mm long × 10 mm wide, were produced. Other studies have only included two different thicknesses (1.5–2 mm) [8,10,11], while others have produced specimens of only one thickness [12,17,18]. In this study, the distance between machine supports to carry out the test was 20 mm, as in other studies [12,19]. Other support spacings used were 24 mm [8,11] and 10 mm [13]. Regarding the number of specimens prepared, although the minimum established is 5, in this case, 10 specimens of each material and each thickness were prepared in order to obtain greater precision in the study, as in Haselton et al. [13] and Canals [11]. Other authors used eight specimens for each study group [20] or even twelve specimens [17].

The materials available for the study were used immediately after the manufacturing process, thus minimizing any type of error that could occur due to inadequate storage conditions, as occurs in the work of Niem et al. [19], where they stated that Structur 3 and Structur Premium suffered problems in their optimum properties due to having too wide a range of storage temperatures. For the storage of the samples, this research chose to preserve them in artificial saliva at 37 ± 1 °C for 28 days. Authors such as Sezin M et al. [20] also preserved their samples at the same temperature for 30 days. Ruttermann et al. [21], Singh et al. [17], and Poonacha et al. [14] also opted for preservation of these in an aqueous or salivary medium. In addition, as in this study, in Zafra [8] and Haselton et al. [13], the samples were also preserved in artificial saliva and subsequently in an incubator at 37 °C. Other authors preserved the samples in distilled water at 37 °C [16]. It should be noted that there is no well-established and standardized protocol for the preservation of the samples, which may mean that different preservation means and times could result in different results.

The results were very positive; however, more studies could be carried out with a greater number of samples and with conditions as close as possible to reality, as these are able to obtain much more exact and truthful results.

In addition, due to the growing demand by patients and professionals for minimally invasive dentistry by complementing it with techniques such as BOPT, there are still many studies that could be developed about this new technique, and that is why sometimes the information found, although valid, was somewhat scarce, requiring more research.

New studies can be developed looking forward to any progress or suggestions on the subject and to be able to address them.

In the present study, flexural strength values ranging from 75.55 to 130.82 MPa were obtained depending on the material and thickness. The lowest value corresponds to the mean value of the Structur 3 specimens at a thickness of 1 mm, while the highest value corresponds to the mean value of the Structur Premium specimens at a thickness of 1.5 mm. Regarding the modulus of elasticity, figures ranged from 2069.28 MPa for the Structur 3 samples at 1.5 mm thickness to 4080.12 MPa for the Structur Premium samples at 1.5 mm thickness.

A study by Bauer et al. [22] provided the results that would be expected for both parameters according to the manufacturer for Structur Premium, which has a flexural strength of 140 MPa and a modulus of elasticity of 2.5 GPa.

It is convenient to highlight the different results obtained by different studies that have either used the same materials and/or carried out the same three-point bending strength test with materials also used as temporary prostheses, thus determining the bending strength and modulus of elasticity.

Values of 104.20 MPa, 95.58 MPa, and 79.54 MPa were obtained in the flexural strength study carried out by Digholkar et al. [12] for three different groups of temporary materials. The first corresponds to PMMA with conventional processing technique, the second group is PMMA processed with CAD-CAM technology, and finally, the lowest value of flexural strength was obtained by the group of resin processed with 3D printing technique. Haselton et al. [13] performed a study to determine the flexural strength of 13 temporary materials after 10 days immersed in artificial saliva. The range of figures oscillated between 56.2–123.6 MPa, with the highest figure corresponding to Provipont and the lowest to Unifast.

In a study by Canals [11], the performance of polylactic acid in terms of marginal fit and flexural strength was studied. Specimens of 1.5 and 2 mm thickness were used, obtaining results for the modulus of elasticity of 3446.0 MPa and 3312.0 MPa, respectively. They concluded that the thicker the material, the greater the force necessary to produce a permanent deformation of the material, with differences found between both thicknesses.

Zafra [8], in a study carried out to know the properties of bisacrylic materials, such as Structur 3, Protemp 4, and Unifast 3, obtained flexural strength values of 70.30 MPa, 95.20 MPa, and 56.69 MPa on average, respectively. Relating the flexural strength to the thickness of the material, this study shows that, for Structur 3, the mean for the 1.5 mm thick tablets was 68.89 Mpa, and for the 2 mm thick tablets, it was 71.51 MPa. In Unifast 3, the 1.5 mm thick tablets had an average of 59.64 MPa and the 2 mm thick tablets 54.14 MPa. As for Protemp 4, the values were 105.09 and 85.30 MPa, respectively. Finally, after the materials were subjected to a thermocycling process, it was observed that in the three materials studied, there was a decrease in the bending strength value when comparing the moment T0 (origin) with T180 (thermocycled).

In a work elaborated by Naresh et al. [10], where the elasticity and bending of PEEK (polyetheretheretherketone) are evaluated, samples of 1.5 mm and 2 mm thickness were analyzed. They concluded that there are statistically significant differences with respect to the modulus of elasticity in relation to the thickness of the PEEK specimens. However, since there are no statistically significant differences between thickness and flexural strength, it was asserted that, in the absence of greater precision, the thickness of the PEEK specimens does not affect the flexural strength of the specimens in the same way as in the present study.

Sezin M et al. [20] subjected different high-, medium-, and low-density resins to flexure. The figures obtained after 30 days of preservation oscillated from 124.35 MPa for Grandio resin from Voco to 59.50 MPa for Rok (SDI). With respect to the elastic modulus, the figures at 30 days oscillated from 10.51 GPa for the Grandio material to 2.17 for Wave Flow. In the study by Singh et al. [17], there were no significant differences in flexural strength between all tested materials when compared to each other at a 24 h interval. On the other hand, there was a large decrease in the flexural strength of all materials when they were retested after 8 days. It is possible to compare the results of this study at 8 days with those obtained by us at 28 days. After 8 days of specimen storage, the results obtained are 37.77 MPa for Cooltemp, followed by Luxatemp (36.28 MPa) and Protemp (35.83 MPa). The lowest value corresponds to SC10 (25.41 MPa). Poonacha et al. [14] studied a self-curing bisacrylic resin stored in artificial saliva for 7 days and obtained flexural strength values of 27.2 MPa, values well below those obtained in this study for either of the two self-curing bisacrylic resins.

This study can directly compare its results with those of other studies that have performed the same test with the same material and two of the thicknesses of the present research. Thus, Zafra [8], studying the flexural strength of Structur 3, obtained an average of 68.89 Mpa for the 1.5 mm thick samples and 71.51 Mpa for the 2 mm thick samples. The averages obtained for both thicknesses are higher, obtaining 83.40 MPa at 1.5 mm thickness and 97.02 MPa at 2 mm, leaving, unlike Zafra, 28 days of storage of the samples in artificial saliva.

With respect to flexural strength, another material such as PEEK [10] obtained values of 247.07 MPa for the 1.5 mm samples and 252.72 MPa for the 2 mm thick specimens. As for the modulus of elasticity, the values obtained for the 1.5 mm and 2 mm specimens are 4150.0 MPa and 4746.0 MPa, respectively. These figures are considered higher than those obtained in this study; however, this material has the disadvantage of the indirect manufacturing technique. 

On the other hand, the results, which do not show statistically significant differences in flexural strength between the thicknesses studied, i.e., 1, 1.5, and 2mm thickness of the two materials, invite further studies to test them at even lower thicknesses in order to be able to use them in minimally invasive preparations with acceptable flexural strength.

New studies are invited to use the same materials at lesser thicknesses in order to be able to use them in minimally invasive preparations with acceptable flexural strength.

In subsequent studies, samples could also be produced to more closely resemble the behavior of the materials in the mouth conditions. In addition, the materials could be studied by manufacturing crowns and giving them different angulations in the cervical area, for example, with which to study the resistance to bending in that area.

Finally, for later studies, it would be convenient and interesting to carry out an assay in which a greater number of samples is included to obtain much more exact results.

## 5. Conclusions

Structur Premium shows a significantly higher flexural strength and modulus of elasticity than Structur 3. There are no significant differences between different thicknesses in terms of flexural strength in Structur Premium and Structur 3, but there are significant differences between different thicknesses in terms of modulus of elasticity in Structur 3. No significant differences were found between thicknesses in terms of modulus of elasticity in Structur Premium.

Further studies would be necessary to evaluate the mechanical characteristics of these materials at thicknesses less than 1 mm. Additionally, studies should be aimed at investigating the mechanical characteristics of these materials from a clinical perspective, for example, the influence of the curvature of the crown and the direction of the force exerted.

## Figures and Tables

**Table 1:** Modulus of elasticity vs. thickness (Structur 3).

STRUCTUR 3	Mean	Standard Deviation	p50	p25	p75
**1 mm**	2249.64	1035.05	2144.47	1334.25	3267.32
**1.5 mm**	2069.27	545.40	2200.20	2017.53	2347.17
**2 mm**	2834.05	670.58	3060.03	2596.96	3136.55
**TOTAL**	2384.32	821.33	2350.41	1999.66	3101.89

**Table 2:** Modulus of elasticity vs. thickness (Structur Premium).

STRUCTUR PREMIUM	Mean	Standard Deviation	p50	p25	p75
**1 mm**	3099.37	468.90	2955.66	2864.32	3290.88
**1.5 mm**	4080.12	708.26	4332.43	3507.27	4546.74
**2 mm**	3899.74	589.89	3927.44	3473.78	4105.68
**TOTAL**	3693.07	721.00	3513.16	2984.19	4266.18

**Table 3:** Flexural strength vs. thickness (Structur 3).

STRUCTUR 3	Mean	Standard Deviation	p50	p25	p75
**1 mm**	75.54	25.46	69.75	62.83	91.03
**1.5 mm**	83.40	20.57	92.67	83.42	96.29
**2 mm**	97.02	16.18	97.44	88.99	109.98
**TOTAL**	85.32	22.25	91.83	67.23	98.48

**Table 4:** Flexural strength vs. thickness (Structur Premium).

STRUCTUR PREMIUM	Mean	Standard Deviation	p50	p25	p75
**1 mm**	120.43	12.69	119.23	115.49	126.25
**1.5 mm**	130.82	18.64	133.64	121.62	144.83
**2 mm**	128.19	13.33	125.71	118.19	131.40
**TOTAL**	126.48	15.27	125.71	118.19	135.16
